# The Effects of Viral Infections on the Molecular and Signaling Pathways Involved in the Development of the PAOs

**DOI:** 10.3390/v16081342

**Published:** 2024-08-22

**Authors:** Xiaozhou Liu, Zhengdong Zhao, Xinyu Shi, Yanjun Zong, Yu Sun

**Affiliations:** 1 Department of Otorhinolaryngology, Union Hospital, Tongji Medical College, Huazhong University of Science and Technology, Wuhan 430022, China; 2 Hubei Province Key Laboratory of Oral and Maxillofacial Development and Regeneration, Wuhan 430022, China; 3 Institute of Otorhinolaryngology, Union Hospital, Tongji Medical College, Huazhong University of Science and Technology, Wuhan 430022, China

**Keywords:** virus, hearing, cytomegalovirus, development

## Abstract

Cytomegalovirus infection contributes to 10–30% of congenital hearing loss in children. Vertebrate peripheral auditory organs include the outer, middle, and inner ear. Their development is regulated by multiple signaling pathways. However, most ear diseases due to viral infections are due to congenital infections and reactivation and affect healthy adults to a lesser extent. This may be due to the fact that viral infections affect signaling pathways that are important for the development of peripheral hearing organs. Therefore, an in-depth understanding of the relationship between viral infections and the signaling pathways involved in the development of peripheral hearing organs is important for the prevention and treatment of ear diseases. In this review, we summarize the effects of viruses on signaling pathways and signaling molecules in the development of peripheral auditory organs.

## 1. Introduction

The vertebrate peripheral auditory organs (PAOs) of vertebrates include the outer, middle, and inner ear. The outer ear is composed primarily of the auricle and external auditory canal, which serves to transmit sound to the eardrum. The middle ear is comprised of various structures, including the tympanic chamber, eustachian tube, sinus, and mastoid, which contain the auditory ossicles. The middle ear transmits sound vibrations to the inner ear. The inner ear is located in the rocky part of the temporal bone and consists of two parts, the vestibule and the cochlea, which provide sensory information about sound, movement, balance, and spatial orientation. Viral infections can affect the development of PAOs and/or damage PAOs, causing hearing loss and balance disorders. Wiertsema et al. [1] identified the presence of human rhinovirus, bocavirus, adenovirus, parainfluenza virus, and respiratory syncytial virus nucleic acids in the nasopharynx of patients with a history of acute otitis media episodes. Vestibular neuritis is believed to have a potential causal link with herpes simplex virus type 1 (HSV-1) infection and reactivation [2]. Pyykko and colleagues [3] conducted serological studies of virus-specific IgG in patients with Meniere’s disease, recurrent vertigo of unknown etiology, and sensorineural deafness. Their findings revealed that these patients exhibited significantly higher titers of varicella zoster, influenza virus B, coxsackievirus B5, and respiratory syncytial virus. Additionally, researchers have identified the presence of parotid and herpes viruses in individuals diagnosed with idiopathic sensorineural sudden deafness [4,5]. Rosenthal et al. [6] followed up 580 neonates with Congenital cytomegalovirus (cCMV) infection and found that 77 had hearing loss at birth, 38 children had delayed hearing loss at the end of follow-up, and delayed hearing loss was strongly associated with symptomatic Human cytomegalovirus (HCMV) infection at birth. cCMV infection occurs in approximately 1% of live births. The majority of infants are born asymptomatic, while approximately 11% present with microcephaly, mental retardation, seizures, hepatosplenomegaly, petechiae, or jaundice [7,8]. Approximately 10% of infants born asymptomatic subsequently exhibit sensorineural hearing loss (SNHL), mental retardation, and learning disabilities [9,10]. These studies all indicate that viral infection is one of the important causes of hearing loss. However, the pathological mechanism of hearing loss caused by viral infection has not been fully elucidated.

cCMV is the most prevalent virus causing SNHL. SNHL induced by cCMV infection may be present at birth or may occur later in childhood, and the severity of cCMV-related hearing loss ranges from unilateral high-frequency loss to severe bilateral loss. Interestingly, hearing loss is typically not apparent in healthy adults, likely due to their well-developed immunity, but it may also be attributed to the fact that mature PAOs are less prone to developmental abnormalities. As developmental defects or stagnation of the inner ear and its surrounding structures are frequently diagnosed in children with SNHL, researchers speculate that CMV infection may affect the formation of the inner ear structure and function by regulating signaling pathways during the development of PAOs. The development of the inner ear is subtly regulated by several important signaling pathways. Cross-species microarrays have identified seven different known signaling pathways; TGFβ, PAX, Notch, Wnt, NFκB, insulin/IGF1, and AP1 [11]. It is possible that viral infections may affect these pathways, thereby impairing inner ear function.

The exact cause of developmental disorders and hearing loss in PAOs caused by viral infections is unknown. There are few studies on the effects of viral infections on the development of the outer and middle ear. Therefore, in this review, we focus on the interactions between viral infections and inner ear developmental pathways and take HCMV as an example to summarize the effects of HCMV infection on inner ear developmental signaling pathways. It is hoped that this knowledge will be useful in the treatment of developmental defects and hearing loss in PAOs and other related diseases caused by viral infections.

## 2. The Development Process of Inner Ear

During organogenesis, signaling pathways between embryonic tissues interact to build highly organized functional tissues and organs. The inner ear contains auditory and vestibular sensory organs. In mammals, the cochlea is responsible for hearing and contains the Corti apparatus, in which mechanosensory hair cells convert acoustic stimuli and generate electrochemical signals in response, which are transmitted to the brain by ear neurons [12]. The vestibular system contains balance receptors for mechanoreceptor hair cells [13]. The enlarged part at the base of the semicircular canals, designated as the juxtaglomerular ridge, is responsible for balance perception. In contrast, the two “maculae” of the globus pallidus and the ellipsoid capsule detect linear and angular acceleration.

**Figure 1:**
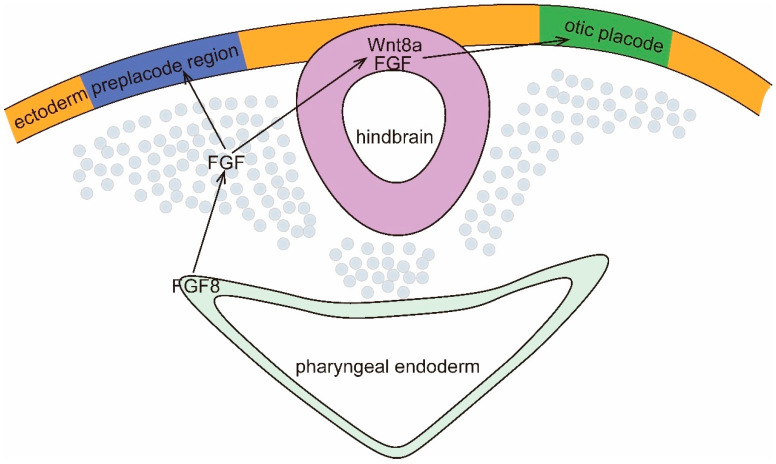
Signals that regulate otic plate (OP) formation. Secretion of FGF8 by the pharyngeal endoderm induces secretion of FGF by the mesoderm near the hindbrain, and FGF defines the posterior PPR (blue) and induces expression of Wnt8a and FGF in the hindbrain. Anterior and posterior hindbrain-derived Wnt8a and FGF-mediated signaling designates the PPR as OP (green).

The sensory organs of the inner ear originate from the otic plate (OP), a thickening of the ectoderm that develops near the hindbrain from the cranial ectoderm immediately lateral to the neural crest (Figure 1). Before the visible morphology of the OP, four genes, namely, Pax2, Sox3, BMP7, and Notch, are expressed in the ectoderm [14]. Subsequently, the ectodermal cell surface undergoes the induction of transcription factors such as the Dlx family, Sox9a, and Foxi1 [15,16]. Fibroblast growth factors produced by the underlying mesenchyme (e.g., FGF10, FGF19, or FGF15) and FGF3 secreted by the hindbrain are also induced [17,18,19]. Following induction, the OP invaginates, separates from the ectoderm, and develops into an ES through interactions with neighboring tissues and the addition of cells from the neural crest and mesoderm [20]. As development progresses, the otic capsule undergoes a transformation from a simple epithelial capsule to a complex fluid-filled labyrinth [21]. The epithelial cells in the anterior medial part of the OP/otic capsule differentiate into a proto-neurosensory epithelium, giving rise to neuromasts, which later form the auditory vestibular ganglion (AVG) [22]. The presensory domain of the OP develops into the Corti apparatus and vestibular sensory organs. The Corti apparatus is a highly specialized acoustic sensory epithelium consisting of sensory hair cells and supporting cells, including outer hair cells, inner hair cells, inner finger cells, Deiters cells, and column cells. The dorsal epithelium of the otic capsule expands to create a vertical capsule. In the vertical outer pouch, opposing epithelial cells come together to form two fusion plates, which subsequently merge and are absorbed to give rise to the two upper semicircular canals and the common peduncle [23,24]. The lateral semicircular canals develop from the lateral sac. Neuronal cells from the ES form the AVG, which contains the neural precursors of the auditory and vestibular ganglia [25]. These ganglia initially form a single ganglion in early development. Ear neurons connect the sensory epithelium to the nucleus accumbens via extensions of the eighth pair of cranial nerves [26]. Concurrently, nearby mesenchymal cells are recruited by the inner ear to form the bony capsule surrounding the labyrinth.

## 3. The Effects of Viral Infections on Hearing

Multiple viruses may infect and affect the development and function of PAOs, including HCMV, HSV, Mumps virus, Epstein–Barr virus, Zika virus, Influenza virus, etc. [27]. cCMV infection is the most common cause of congenital deafness in children [28]. Almost 60% of symptomatic infants with CMV infection will suffer from permanent sequelae, with SNHL being the most common abnormality. Mumps virus may be a cause, accounting for about 7% of adults with sudden hearing loss [29]. Coscia et al. [30] evaluated 63 children with cCMV infection by auditory brainstem responses (ABRs) and found that 20 ears showed varying degrees of hearing loss and that 4 of these ears worsened from moderate to severe hearing loss. Ronner et al. [31] tested 530 infants who failed newborn hearing screening for CMV, of whom eight tested positive and six CMV-positive infants demonstrated hearing loss in their ABR. In a study of 1720 infants in India, Dar et al. [32] found that 20 of the 40 children who did not pass the hearing screening were CMV-positive. The ABR confirmed that 11 of these children had unilateral or bilateral permanent congenital or early-onset hearing loss. The phenotype of hearing loss caused by viral infection is diverse and can be mild, moderate, or even profound, unilateral or bilateral, and sudden or progressive. However, the reasons and mechanisms underlying the heterogeneity of phenotypes and asymmetrical hearing loss in patients with cCMV infection still need to be elucidated.

## 4. The Effects of Viral Infections on the Development, Structure and Function of Cochlear

Although it is difficult to obtain pathological specimens of a CMV infection patient’s temporal bone, there are still individual studies reporting pathological damage to the human cochlea caused by viral infection. Tsuprun et al. reported a loss of vestibular hair cells, outer hair cells in the organ of Corti, and nerve fibers in the area of dark cells in the vestibular labyrinth of the different temporal bones of newborns with cCMV infection. Cytomegalic cells in both cochlear and vestibular parts of the inner ear, hypervascularity in the stria vascularis, and large areas of cellular and non-cellular structures were observed in the scala tympani of the perilymphatic space, respectively [33]. Rarey et al. [34] reported temporal bones examined from patients who had died of sequelae of congenital cytomegalic inclusion disease. Endolymphatic hydrops in the basal turn of the cochlear duct and collapsed Reissner’s membranes in the more apical turns were observed. Strial atrophy and a loss of cochlear hair cells were observed along the entire length of the basilar membrane. Vestibular neuroepithelial regions were degenerated and fibrosis was seen within the vestibular perilymphatic tissue spaces. Distention of the saccular membrane was evident. Isolated regions of calcifications were observed in both cochlear and vestibular tissues [34]. Due to the difficulty in obtaining pathological specimens of the CMV infection patient’s temporal bones, viruses are commonly injected into the cerebral cortex, ventricles, middle ear, or scala media of the cochlea in animals to study the cochlear pathological damage and hearing loss mechanisms caused by viral infection. Almishaal et al. [35] reported a reduction in the wave I amplitude of ABR and synaptic counts before progressive hearing loss and hair cell damage in mouse cytomegalovirus (MCMV)-infected mice, which suggests that the developmental disorders and structural damage of the cochlea caused by MCMV infection may be the cause of hearing loss. Carraro et al. [36] reported vascular damage after MCMV migration to the inner ear, which suggests that initial auditory threshold losses may relate to the poor development or maintenance of the endocochlear potential and supply of energy caused by strial dysfunction. Nomura et al. [37] inoculated guinea pig cytomegalovirus (GPCMV) and HSV-1 into the middle ear of guinea pigs and observed the presence of GPCMV inclusion body-bearing cells within the cochlea, which also provided evidence that virus can indirectly infect the cells of PAOs. The pathological changes in the inner ear caused by MCMV infection could be sustained. Tian et al. observed pathological changes in the inner ear at different times in mice injected with MCMV in the brain. After infection, hemorrhage of scala tympani and scala vestibule appeared and reached the highest peak after 3 days, accompanied by inflammatory cell infiltration; the vestibular membrane thickened after 5 days; the cell gaps of SGN cells widened and were arranged more sparsely with cell edema after infection for 7 days, accompanied by the infiltration of plasma cells; and fibroblast proliferation and fibrosis appeared after infection for 14 days [38].

The tectorial membrane of guinea pig inoculates with HSV injected into the scala tympani showed various morphological changes like atrophy, roll-up, and dot formation, which are similar to the various morphological changes in the tectorial membranes of both temporal bones of patients who suffered from sudden deafness [39]. Hearing loss and vestibular dysfunction were also observed in all mice after inoculation of HSV-1 and HSV-2, while the HSV antigen was detected in vascular striations and vestibular ganglion cells [40]. Part of the infected columnar epithelial cells in the stria vascularis underwent apoptosis, while many uninfected cells in the spiral organ of Corti were apoptotic. Vestibular ganglion cells were largely infected, but not apoptotic, which suggested that the vestibular dysfunction occurring in CMV-infected patients may be related to the vestibular ganglion cells’ abnormal function in vestibular nerve cells instead of cell death. The narrowing of reticular tissue spaces caused by swelling of the periotic duct tissue and complete obstruction of the cribriform structure in the internal orifice of the cochlear aqueduct were observed in guinea pigs inoculated with HSV-1 in cisterna magna [41]. These studies provided evidence that virus infection can affect the development, structure, and function of PAOs. It should be noted that most researchers construct animal models of hearing loss after viral infection by injecting viruses into the brain. The virus may first infect the auditory cortex rather than the PAOs, which may have an impact on the occurrence of hearing loss and pathological damage to the cochlea.

## 5. The Effects of Viral Infections on the Development, Structure, and Function of Ossicular Chains

Mammalian ossicular chains contain three auditory ossicles: the malleus, incus, and stapes. The auditory ossicles are mainly derived from cranial neural crest cells (NCCs). NCCs migrating to the first pharyngeal arch (PA1) form the malleus–incus condensation [42]. In mouse PA1, the malleus–incus forms as a single cohesive mass attached to Meckel’s cartilage at embryonic day (E) 10.5. Subsequently, the malleus and incus bones separate from each other at E13.5 [43,44]. NCCs migrating to the second pharyngeal arch (PA2) form the stapedial condensation [45]. At E10.5, the stapedial condensation forms separately in the PA2 mesenchyme [46]. The stapes acquires its characteristic stirrup-like structure at E11.5. The stapes is attached to the inner ear by annular ligaments at E15.5.

Sonic Hedgehog (SHH), bone morphogenetic protein 4 (BMP4), FGF, and Notch signaling molecules play a crucial role in middle ear development. Shh is expressed in the endoderm of PA1 at E10.5 and its receptor is expressed in the mesenchyme adjacent to the endoderm, forming the malleus–incus anlagen. Knockout of Shh in the pharyngeal endoderm results in a loss of malleus–incus condensation in PA1 [42]. The forced activation of the SHH signaling pathway in NCCs impairs the development of normal auditory ossicles [42]. BMP signaling mediates the induction and migration of NCCs [47]. Mouse embryos with downregulated expression of BMP signaling in NCCs exhibit the loss of stapes and the styloid process [48]. The knockdown of Bmp4 in the pharyngeal endoderm results in the disruption of NCC migration to the stapes region in PA2, which in turn affects the formation of the stapes [42]. Fgf8 is strongly expressed in the arch ectoderm and the pharyngeal endoderm [49]. Fgf8-deficient mice exhibit severe deficits in the malleus and incus bones [50]. Heterozygotes with mutations in FGF receptor 1 show incus abnormalities and multiple malformations of the stapes bone [51]. Mice lacking Jag1 or Notch2 in NCCs show malformed stapes, manifesting as a monopodial structure lacking crural regions [52].

Many reports have described the presence of measles virus (MV) RNA or protein in the ear bones of patients with otosclerosis [53,54,55]. Therefore, researchers hypothesized that persistent MV infection of the ES is one of the causes of otosclerosis. The characteristic pathological change in otosclerosis is a disturbed reconstruction of the bone of the ear capsule. This abnormal bone reconstruction can lead to stapes fixation, resulting in conductive hearing loss, and can also be combined with SNHL [56]. Furthermore, immunohistochemical staining of the fragments of osteosclerotic footplates revealed the expression of mumps, MV, and rubella virus antigens in vascular connective tissue, osteoblasts, osteoclasts, and chondrocytes in or around the lesion area [57]. These results support the idea that otosclerosis is caused by a viral infection. However, how the viral infection affects the development of the ossicles of the ear capsule remains unclear, and more research is needed to elucidate the underlying mechanisms.

## 6. Programmed Cell Death Pathways and Inflammation Signaling Pathway

Damage to the cochlear cell induced the chemotaxis of infiltrating immune cell types and the release of cytokines, as well as generating reactive oxygen species (ROS) and cytokines, leading to irreparable damage and loss to hair cells and neurons. Qiao et al. [58] reported that intracellular Ca^2+^ levels and CaM mRNA levels increased in the cochlear neurons of mice intracranial inoculation with MCMV, while the mitochondrial membrane potential decreased. These reports suggest that MCMV may cause sensorineural hearing loss by affecting the signaling pathway regulating intracellular Ca^2+^ concentration. The disorder of intracellular Ca^2+^ concentration and abnormal mitochondrial membrane potential may further lead to energy supply disorders and ROS accumulation, which may initiate the process of cell apoptosis. Li et al. [59] reported that the B-cell lymphoma 2(Bcl-2) and B-cell lymphoma 2-associated(Bax) protein ratio was decreased in spiral ganglion neurons of mice intracranially injected with MCMV, which suggested that the initiation of the apoptotic signaling pathway regulated by MCMV in spiral ganglion neurons is an important component of the SNHL mechanism in infected mice. In addition, pyroptosis and apoptosis are also involved in CMV-induced SGN death and are mainly regulated by activated caspase-1, caspase-8, and NLRP3, which suggested that MCMV-induced SGN programmed death may be related to inflammation responses [60,61]. Schachtele et al. [62] reported that MCMV-infected cochlea revealed a robust and chronic inflammatory response with a prolonged increase in reactive oxygen species production by infiltrating macrophages. Bradford et al. [63] identified that viral antigens were present in the inner ear, as were CD^3+^ mononuclear cells in the spiral ganglion and stripe vasculitis of MCMV-infected mice. Qiao et al. [64,65] also observed increased levels of lymphocytic infiltration in the membrana vestibulitis, high levels of TNF-α and IL-6 in scala tympani, and increased levels of downstream inflammatory factors IL-1β and IL-18 in a mouse model of intracranial inoculation with MCMV. The deletion of a specific viral immunomodulatory gene, macrophage inflammatory protein (MIP) 1alpha homolog, could improve the hearing of MCMV-infected mice [66]. These studies suggested that the chemotaxis and polarization of macrophages may be the reason for the loss of hair cells and spiral ganglion cells after MCMV infection [67].

Harding et al. [68] reported RNA sequencing of otic progenitor cells (OPCs) from hiPSC with Zika virus and cytomegalovirus infections. ZIKV infection rapidly and significantly induces the expression of type I interferon and interferon-stimulated genes, which may contribute to OPC apoptosis. Several transcripts of key stress/apoptosis-associated genes, including DDIT3 (CHOP) and PMAIP1 (NOXA), were regulated, while the chemokine CCL5 (RANTES) was differentially upregulated in ZIKV-infected OPCs. In contrast, interferon-stimulated and inflammatory genes did not display upregulation in HCMV-infected OPCs, nor apoptosis or stress-associated genes. CCL5 levels were elevated in HCMV-infected OPCs. CCL2 levels, which may be associated with neuronal degeneration, were downregulated in both ZIKV- and HCMV-infected OPCs. Interleukin-18 (IL-18) levels were also increased in HCMV-infected OPCs. These studies suggest that there are differences in the regulation of signaling pathways after different viruses infect PAOs.

## 7. Virus Infection Regulates Sox2: A Transcription Factor Required for Inner Ear Growth and Cochlear Nonsensory Formation

Sox2, a member of the Sox B1 family of transcription factors, directs the differentiation of pluripotent stem cells into neural progenitor cells (NPCs) and the maintenance of neural progenitor stem cell identity [69]. Genome-wide analysis showed that HCMV-infected NPCs exhibited the downregulation of Sox2 [70]. Wu et al. [71] found that the HCMV immediate early protein 1 (IE1) mediated Sox2 protein depletion in infected NPCs by promoting nuclear accumulation and inhibiting the phosphorylation of STAT3 to cause Sox2 downregulation, which suggested that HCMV infection may be involved in organ development by altering cell fate decisions by perturbing the SOX2 pathway.

Sox2 missense or heterozygous loss-of-function mutations have been shown to cause bilateral anophthalmia, which can be accompanied by learning disabilities, seizures, brain malformations, or hearing loss [72]. In Sox2-deficient mice, inner ear neurons initially form normally, whereas late-differentiating neurons in the cochlear apex are never formed, and there is a complete absence of sensory epithelium formation in the apical part of the juxtaposition of the semicircular canals and in all three cristae [73]. In addition, the transcription factor Sox2 is a key gene for cochlear hair cell development. Sox2 activates the transcription factor Atoh1, which is important for hair cell differentiation, by interacting with the 3′ enhancer of Atoh1 [74]. The deletion of Sox2 results in impaired inner ear development with reduced and disorganized hair cells [75]. Hair cell development was found to cease even after progenitor cells were fully established in mice conditionally knocked out of Sox2, and silencing also inhibited postnatal differentiation of hair cells induced by the inhibition of γ-secretase [76].

## 8. Virus Infection Regulates the Wnt Signaling Pathway: Affecting Auditory Substrate Specialization, Otic Vesicle Formation, and Hair Cell Differentiation

**Figure 2:**
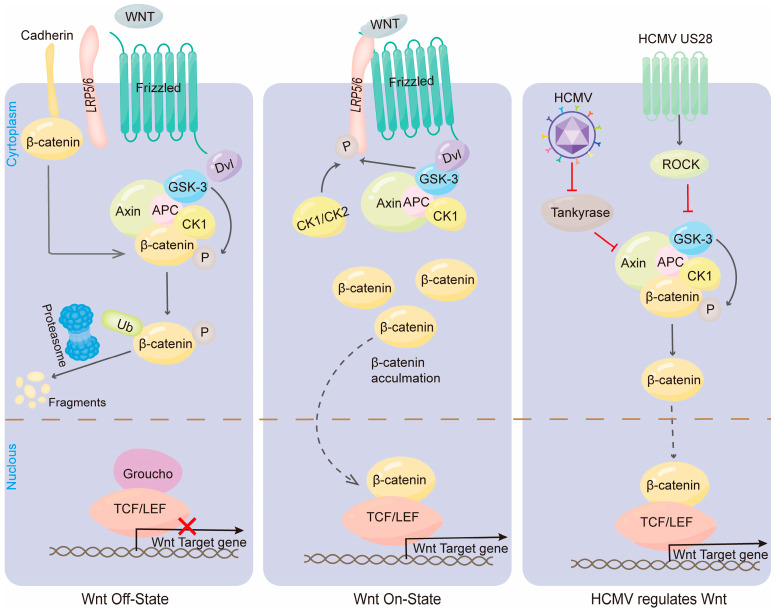
HCMV regulates the classical Wnt/β-catenin signaling pathway. When the Wnt pathway is switched off (the Wnt receptor complex is not bound to the ligand), GSK3α/β phosphorylates β-catenin, which is then ubiquitinated and rapidly destroyed by proteasome targeting. In the nucleus, binding of Groucho to TCF/LEF inhibits transcription of Wnt target genes. When the Wnt signaling pathway is opened, the Fzd/LRP receptor complex activates the classical signaling pathway. Receptor recruitment to Dvl serves as a docking platform for components of the β-catenin disruption complex. Wnt induces GSK3β and CK1/CK2 to phosphorylate LRP5/6, which regulates Axin docking and releases β-catenin. In the nucleus, β-catenin replaces Groucho in Tcf/Lef, thereby promoting transcription of Wnt target genes. HCMV reduces the poly-ADP-ribosylation activity of end-anchor polymerase, thereby stabilizing Axin and inhibiting the Wnt pathway. HCMV US28 inhibits GSK3 activity via the ROCK pathway, β-catenin cannot be phosphorylated for degradation HCMV US28 inhibits GSK3 activity through the ROCK pathway, and β-catenin cannot be phosphorylated and degraded.

The Wnt signaling pathway is a highly conserved mechanism that plays a crucial role in various developmental processes, including cell fate determination, cell migration, neural patterning, and cell polarity [77]. This binding of Wnt proteins to the extracellular surface initiates the activation of intracellular signaling pathways, such as the classical Wnt pathway, the non-classical planar cell polarity (PCP) pathway, and the non-classical Wnt/calcium pathway (Figure 2) [78]. The PCP pathway regulates the orientation of hair cell cilia bundles and the convergent extension of the cochlea during cochlear tube formation.

HCMV can target the Wnt pathway and regulate its activity. Stevens et al. [79] employed a retroviral gene to stimulate the Wnt signaling pathway, with the aim of modifying the morphogenesis of the chicken inner ear, including ectopic and fused sensory patches, which may be caused by the misexpression of Wnt3a. Teo et al. [80] observed increased expression levels of Wnt11, Fzd7, GSK3β, and β-catenin in the Wnt signaling pathway during HCMV infection in HCMV-infected colorectal cells and their derived cells. Zhou and colleagues [81] found that Mouse cytomegalovirus (MCMV) infection significantly inhibits the expression of Wnt-1 in NSCs cultured in vitro, which affects the differentiation of NSCs. HCMV can also negatively regulate the Wnt pathway. Angelova et al. [82] demonstrated that HCMV disrupts Wnt/β-catenin signaling in dermal fibroblasts and human placental trophoblasts. HCMV infection alters the subcellular distribution of β-catenin, with decreased levels of membrane-associated and cytoplasmic pool β-catenin, and the accumulation in discrete nuclear regions, inhibiting Wnt/β-catenin signaling. Roy et al. [83] demonstrated that HCMV infection inhibits the PARsylation activity of Tankyrase, leading to the accumulation of Axin1 (a negative regulator of the Wnt pathway) and a reduction in its PARylation, thereby inhibiting the β-catenin pathway. It is not yet clear whether HCMV affects the non-canonical Wnt signaling pathway. However, Zuylen et al. [84] demonstrated that HCMV infection can increase the expression of the non-canonical Wnt receptor ROR2 to alter Wnt5a-mediated signaling and modulate the migration of nourishing cells.

The classical Wnt/β-catenin signaling pathway is particularly important for ear development. Cochlear development in selective knockout Wnt5a mice showed significant PCP defects, as evidenced by disorganized hair cell orientation and shorter cochlea formation compared to wild-type mice [85]. The Wnt/β-catenin pathway plays a crucial role in various processes during inner ear development, such as the specialization of the auditory substrate, the formation of auditory vesicles, and the regulation of hair cell differentiation [86]. Jacques et al. [87] applied FH535, an inhibitor of the Wnt/ β-catenin signaling pathway, to cochlear explants at Embryonic day 12.5, resulting in the inhibition of the Wnt/β-catenin signaling pathway, and the results showed that the differentiation of sensory progenitor cells to hair cells was inhibited. Conversely, the addition of LiCl (a Wnt signaling activator that prevents the degradation of β-catenin by inhibiting GSK3β activity) [88] activated the Wnt/β-catenin pathway and led to an increase in the number of differentiated hair cells.

Many studies have confirmed that HCMV can regulate the Wnt pathway. However, the impact of HCMV infection on the regulation of the Wnt pathway in inner ear development remains unclear, and further research is needed to elucidate its potential mechanisms.

## 9. Virus Infection Regulates the Notch Signaling Pathway: Affecting Cell Fate and Interfering with Inner Ear Development

**Figure 3:**
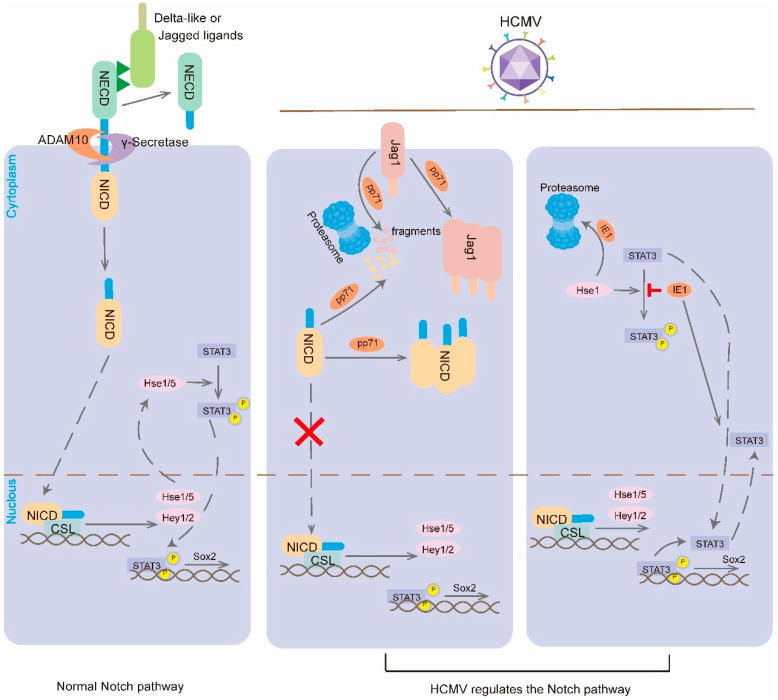
HCMV regulates the Notch signaling pathway. When Notch receptor binds to Notch ligand, Notch receptor is cleaved by ADAM10 and γ-secretase, leading to the release of Notch intracellular structural domain (NICD). NICD fragment is the active form of the receptor that binds to the CSL transcription factor to trigger the transcription of the Notch target genes. Hes proteins also promote the phosphorylation and activation of STAT3, and the activated STAT3 shuttles to the nucleus to bind the Sox2 promoter and induce its expression. HCMV is a membrane protein that regulates Notch signaling pathway phosphorylation and activation and activated STAT3 shuttles to the nucleus to bind the Sox2 promoter and induce its expression. HCMV periplasmic protein pp71 can alter the cellular localization of Jag1 and NICD and inhibit Notch signaling by promoting their degradation via the proteasome. HCMV IE1 promotes proteasomal degradation of Hes1 to inhibit the phosphorylation of STAT3 and promotes the accumulation of unphosphorylated STAT3 in the nucleus and inhibits Sox2 transcription.

The Notch pathway is highly conserved throughout evolution and is considered to be one of the major signaling pathways coordinating the developmental processes of most organs and tissues in all postnatal animals (Figure 3) [89]. It is involved in the coordination between neighboring cells during development and homeostasis [90].

HCMV infection can interfere with organ development by altering cell fate decisions through disruption of the Notch pathway. In HCMV-infected NPCs, the expression of the periplasmid proteins pp71 and UL26, both of which are expressed endogenously and exogenously, results in a reduction in the levels of NICD1 and Jag1 proteins and an alteration in the subcellular localization of NICD1 [91]. The mRNA levels of Notch-related receptors and ligands were found to be significantly reduced in HCMV-infected NSCs following 1 day of induced differentiation. In particular, the levels of Notch1, Notch2, and DLL1 were found to be significantly decreased. Furthermore, the intracellular levels of NICD were found to be significantly reduced 7 days after viral infection. HCMV infection also promotes the proliferation of U251 glioma cells by regulating the ATF5 signaling pathway to upregulate the expression of NICD and Notch1 [92]. Hes1 serves as an important downstream effector of the Notch signaling pathway, and deletion of the Hes1 protein inhibits NPC proliferation and neutrosphere formation, driving aberrant differentiation of NPCs. Liu et al. [93] were the first to observe that HCMV infection disrupts the Hes1 rhythm to interfere with NPCs cell fate. Further studies revealed that IE1 of HCMV promotes Hes1 ubiquitination and degrades Hes1 through the proteasome, downregulating Hes1 expression [94].

The Notch signaling pathway regulates the proliferation of inner ear sensory pre-cursor cells and maintains the homeostasis of cochlear sensory epithelial cell number and structure through lateral inhibition and lateral induction [95]. In the initial stages of inner ear development, there is a positive feedback loop between the Wnt and Notch pathways, which together coordinate the refinement of the auricular plate boundary. The Wnt pathway regulates the early expression of Notch1, Jag1, Hes1, and Hey1 in the auricular plate. The Notch pathway also regulates the expression of ear markers such as Pax8 and the thickening of the ear plate. The inactivation of Notch1 reduces the size of the ear plate. Although Notch signaling does not regulate its own expression in the auricular plate and the onset of activation, it enhances Wnt activity, thereby maintaining Notch activity [96].

The Notch pathway also inhibits lateral inhibition, thus forming a mosaic pattern of hair/support cells. Activated Notch receptors inhibit the expression of Jag2, Dll1, and Atoh1 in signaling cells, resulting in the differentiation of signaling cells into supporting cells and adjacent cells into hair cells.

## 10. Other Signaling Pathways Regulated by HCMV

Yee et al. [97] reported that ZIKV infection alters the cochlear expression of genes that signal cell damage (S100B), transport fluids (AQP1), function as gaseous transmitters (eNOs), and modulate immune response (F4/80). Harding et al. reported that protocadherin SEMA3C and SEMA3F, which belong to the semaphorin class 3 family of neuronal guidance genes transcript levels, were downregulated. These results suggest that ZIKV infection may reduce neuronal maturation or synapse formation in PAOs. In addition, the expression of EML1 associated with hearing loss in Usher syndrome type 1 and CXCL12 associated with cellular movement and angiogenesis induction were also downregulated [68]. HCMV infection disrupted the expression of key genes and pathways associated with inner ear development and function, including Cochlin, nerve growth factor receptor, SRY-box transcription factor 11, and transforming growth factor-beta signaling, instead of upregulating interferons or causing cell viability reduction. Insulin growth factor binding proteins 3 and 5 (IGFBP3 and IGFBP5), neuronal regeneration-related protein (NREP), SOX11, LRRC32, ITGAV, ITGβ8, and TGFβ were also downregulated in HCMV-infected OPCs, which may be a signal that alters neurovascular development and leads to neurodegeneration.

Experiments conducted in cells derived from organs or tissues other than the cochlea have shown that HCMV can also regulate other signaling pathways related to development. HCMV can target Pax2, and in primary fibroblasts infected with HCMV, Browne et al. applied gene chip technology analysis to find that PAX2 was downregulated at 1- and 48-h post-infection [98]. The HCMV-induced downregulation of PAX2 may hypothetically disrupt the otic medial–lateral specification toward the generation of neuroblasts within the epithelium of otocyst, which subsequently affects the segregation and migration of vestibular and auditory neuromasts from the neurosensory domain to the vestibular cochlear ganglion. Similarly, HCMV infection in fibroblasts also downregulates the transcription factor SIX1, which plays a role in the axial specification of ES [98]. MCMV-infected differentiated mouse NSC significantly downregulates the transcription factor NGN1, which determines vestibulocochlear ganglion nerve fate [81,98]. HCMV major IE1 can interact with FGFR3 in astrocytoma cell lines, but the absence of FGFR3 leads to abnormal column cell development [99,100]. Furthermore, FGFR3-deficient mice exhibit deficiencies in supporting cell differentiation, and a virus-induced excess of FGFR3 may alter the balance of sensory and non-sensory cells [101]. Another possibility for hearing damage from HCMV infection is reduced levels of CDKN1B, an enzyme inhibitor that regulates the G1 phase of the cell cycle [98]. HCMV infection of human embryonic lung cells leads to CDKN1B degradation [102]. The deletion of CDKN1B results in the overproduction of hair cells and supporting cells during cochlear development, which in turn causes severe hearing loss [103].

## 11. Conclusions

Viral infections are considered a significant cause of congenital hearing loss, but little attention has been paid so far to the effects of viral infections on the molecular and signaling pathways involved in the development of PAOs. The aim of this paper was to summarize the effects of viruses (especially HCMV) on the main signaling molecules and signaling pathways involved in PAO development. Regulating the signaling pathways of PAO development can play a therapeutic role in related diseases. Inhibitors of the Wnt pathway have been demonstrated to suppress the replication of HCMV and the expression of viral proteins IE2, UL44, and pp65 [104]. Notch signaling blockers also show favorable effects on the recovery of hearing loss in mice [105]. The inhibitor of γ-secretase induces new hair cells and partially restores noise-induced hearing loss [106]. We hope that the study of abnormalities in the developmental pathway of PAOs caused by viral infection will provide new directions for intervention in virus-mediated developmental malformations of PAOs and related diseases.
